# Implementation strategies for cancer rehabilitation in clinical practice: a Delphi study

**DOI:** 10.1093/jjco/hyag085

**Published:** 2026-06-10

**Authors:** Nanako Hijikata, Nobuko Konishi, Noriatsu Tatematsu, Nanako Nishiyama, Kazuhiro Kojima, Daisuke Ito, Yoshitada Sakai, Ken Kouda, Akira Tanuma, Yasuyuki Takakura, Junichiro Inoue, Takeshi Kobayashi, Takuro Sakurai, Noriyo Sugimori, Kyoko Abe, Miho Kurihara, Tetsuya Tsuji

**Affiliations:** Department of Rehabilitation Medicine, Keio University School of Medicine, Shinjuku-ku, Tokyo 160-8582, Japan; Department of Rehabilitation Medicine, National Cancer Center Hospital East, Kashiwa-shi, Chiba 277-8577, Japan; Department of Integrated Health Sciences, Nagoya University Graduate School of Medicine, Nagoya-shi, Aichi 461-8673, Japan; Graduate School of Rehabilitation Science, Osaka Metropolitan University, Osaka-shi, Osaka 536-8525, Japan; Department of Rehabilitation Medicine, Keio University Hospital, Shinjuku-ku, Tokyo 160-8582, Japan; Department of Rehabilitation Medicine, Keio University School of Medicine, Shinjuku-ku, Tokyo 160-8582, Japan; Division of Rehabilitation Medicine, Kobe University Graduate School of Medicine, Kobe-shi, Hyogo 650-0017, Japan; Department of Rehabilitation Medicine, Wakayama Medical University, Wakayama-shi, Wakayama 641-8509, Japan; Department of Rehabilitation Medicine, Juntendo University Shizuoka Hospital, Izunokuni-shi, Shizuoka 410-2295, Japan; Department of Physical Therapy, Faculty of Health and Medical Care, Saitama Medical University, Moroyama, Saitama 350-0496, Japan; Division of Rehabilitation Medicine, Kobe University Hospital, Kobe-shi, Hyogo 650-0017, Japan; Department of Occupational Therapy, Nihon Institute of Medical Science, Iruma-gun, Saitama 350-0435, Japan; Rehabilitation Unit, National Cancer Center Japan Hospital, Chuo-ku, Tokyo 104-0045, Japan; Department of Rehabilitation Medicine, Tokyo Medical University Hospital, Shinjuku-ku, Tokyo 160-0023, Japan; Department of Clinical Nursing, Chiba Faculty of Nursing, Tokyo Healthcare University, Funabashi-shi, Chiba 273-0027, Japan; Department of Nursing, National Cancer Center Hospital East, Kashiwa-shi, Chiba 277-8577, Japan; Department of Rehabilitation Medicine, Keio University School of Medicine, Shinjuku-ku, Tokyo 160-8582, Japan

**Keywords:** exercise therapy, implementation science, neoplasm, rehabilitation, survivorship

## Abstract

**Objective:**

The purpose of this study was to identify implementation strategies and determine priorities for establishing a seamless cancer rehabilitation delivery system based on consensus among multidisciplinary stakeholders.

**Methods:**

An online modified-Delphi study was conducted. A total of 100 stakeholders (healthcare professionals, researchers, cancer survivors, and members of patient support groups) with experience in cancer rehabilitation were invited. Participants selected the top three barriers to cancer rehabilitation in the first survey. In the second survey, the implementation strategy list was provided, and the effectiveness of each strategy was rated using a 4-point Likert scale. In the third survey, participants evaluated the impact and cost of successful implementation using a 4-point scale to determine priority.

**Results:**

Valid responses were obtained from 85 participants for the first survey, 64 for the second survey, and 59 for the third survey. Consensus was reached on all 35 implementation strategies as effective measures (agreement rate for each item ranged from 70.3% to 98.4%). Fourteen items were judged very effective. The assessment of priority considering impact and cost indicated that reinforcing multidisciplinary collaboration and improving access to information were high priorities.

**Conclusions:**

This study identified implementation strategies and priorities for cancer rehabilitation based on expert consensus. These findings demonstrate that improving inter- and intra- organizational processes is as crucial as institutional and structural enhancements. The identified strategies are expected to bridge the gap between evidence and clinical practice in Japan, contributing to the promotion of effective, continuous cancer rehabilitation delivery systems.

## Teaser

This study identified implementation strategies and determined priorities for cancer rehabilitation based on expert consensus. These strategies are expected to bridge the gap between evidence and clinical practice in Japan.

## Introduction

Cancer incidence in Japan has continued to increase for both men and women since the 1980s, with approximately one in two people being diagnosed with cancer during their lifetime [1]. Cancer has been the leading cause of death in Japan since 1981, recently accounting for about 30% of all deaths [2]. Conversely, due to advances in medical technology, the age-adjusted mortality rate has been on a downward trend since the late 1990s [2, 3]. The five-year relative survival rate for people diagnosed with cancer between 2009 and 2011 in Japan was 64.1% [4]. Extended survival time is now achievable, shifting the paradigm from an era of cancer as an ‘incurable disease’ to one of ‘living with cancer.’

Cancer patients often experience various functional impairments during cancer therapy, which decrease their quality of life (QOL) due to limitations in activities of daily living (ADL) and instrumental activities of daily living (IADL) [5]. A nationwide questionnaire-based survey of cancer survivors in Japan [6] indicated that over half have cancer-related functional impairments or treatment-related side effects during treatment. After treatment, over half experience reduced endurance, approximately 40% continue to have functional impairments or side effects, and about 20% face difficulties in daily living.

Cancer rehabilitation, aimed at improving and maintaining the functional abilities of cancer patients throughout the entire cancer journey from diagnosis to end-of-life, is playing an increasingly vital role. To address physical decline, guidelines for cancer survivors from the American Cancer Society, American College of Sports Medicine, and other societies recommend at least 150 minutes of moderate-intensity physical activity and two sessions of resistance training per week [7–10]. For elderly patients, physical therapy and/or occupational therapy is recommended when a decline in ADL or IADL is observed [11, 12]. For specific cancer-related functional impairments, impairment-driven rehabilitation is provided by rehabilitation professionals skilled in cancer rehabilitation [13].

In Japan, since the enactment of the Cancer Control Act in 2006, the infrastructure for cancer rehabilitation has been developing, and the ‘Cancer Patient Rehabilitation Fee’ was newly established in the medical fee schedule in 2010. The Third Basic Plan for Cancer Control Promotion (2017–2022) positioned the equalization and consolidation of cancer rehabilitation services as one of its key priorities [14]. The percentage of designated cancer care hospitals and other facilities with rehabilitation professionals increased from 43.9% in 2018 to 51.0% in 2021. The percentage of cancer patients receiving rehabilitation during cancer treatment rose from 19.7% in 2012 to 30.7% in 2016 [15].

Despite efforts centered on designated cancer care hospitals, challenges remain in cancer rehabilitation practice. The shift of cancer treatment from inpatient to outpatient settings is progressing due to the Diagnosis Procedure Combination (DPC) system imposing restrictions on hospitalization periods and the increasing number of cancer patients receiving chemotherapy and/or radiation therapy on an outpatient basis. Consequently, the provision of cancer care in the community is expanding. To establish seamless continuity of cancer rehabilitation after discharge and during outpatient care, developing rehabilitation delivery systems across various medical and care settings is now an urgent priority [16].

To promote the provision of effective and continuous cancer rehabilitation, enabling its implementation across diverse medical and care settings is essential. Successful implementation requires understanding barriers to implementation and using context-specific strategies. Implementation strategies are defined as methods or techniques used to enhance the adaptation, implementation, sustainability, and scaling-up of evidence-based interventions [17–19], bridging the gap between evidence and clinical practice.

This study aimed to identify implementation strategies for establishing a seamless cancer rehabilitation provision system based on consensus among multidisciplinary stakeholders in Japan. A seamless cancer rehabilitation provision system was defined as ‘a system that provides cancer rehabilitation across various medical and care settings, including designated cancer care hospitals, regional medical institutions, convalescent rehabilitation hospitals, hospitals in community-based integrated care, community-based daycare/day services, and home-visit care.’

## Materials and Methods

### Study design

In this study, a modified Delphi method was used to perform an online survey in accordance with international guidance on conducting and reporting of Delphi studies [20–23]. The results of the initial survey were presented in the next round, and re-evaluation was requested based on the results [22]. A maximum of three rounds was set [24]. This study was approved by the institutional ethics review board (No. 20241093).

### Participants

The participants were stakeholders (target N = 100), including healthcare professionals, researchers, cancer survivors, and members of patient support groups, with experience in cancer therapy, cancer rehabilitation, or survivorship support. Participants were selected from members of the Cancer Rehabilitation and Lymphedema Care Network Consortium and of the Cancer Rehabilitation Group in the Japanese Association of Supportive Care in Cancer (JASCC). The Cancer Rehabilitation and Lymphedema Care Network Consortium consists of 54 organizations (including 104 nominating committee members) that connect medical institutions, community-based and home-visit care organizations, and patient support groups, where representativeness across geographic regions and clinical settings is ensured. Invitations were sent via email to nominating committee members from organizations related to cancer rehabilitation (see Supplementary Table 1) and to the JASCC Cancer Rehabilitation Group, and 100 individuals who gave their consent were appointed as panel members. The questionnaires were sent to them, with responses required to be submitted within two weeks; non-responders received a single reminder with a one-week extension. Incomplete responses were excluded from the analysis. The questionnaires used in this study are presented in Supplementary Tables 2–4.

### A survey on barriers to cancer rehabilitation

In the first survey, participants were asked to respond to questions regarding barriers to cancer rehabilitation. They were presented with options describing reasons that constitute barriers to cancer rehabilitation across the following domains: ‘Facilities/equipment, Healthcare professionals, and Medical reimbursement,’ ‘Medical collaboration,’ ‘Rehabilitation goal-setting,’ ‘Risk management,’ and ‘Communication with cancer patients and their families.’ Participants selected the top three options they felt applied the most to each domain. In addition, they rated the importance of each domain in cancer rehabilitation best practices on a five-point scale. The options used in this survey were developed based on a scoping review regarding barriers to cancer rehabilitation in Japan conducted by our research team.

### Development of an implementation strategy list for cancer rehabilitation

In this study, an implementation strategy list was developed to address barriers to cancer rehabilitation. The implementation strategies were based on content mentioned as problem-solving measures in a scoping review of barriers to cancer rehabilitation. Each item was categorized using the Expert Recommendations for Implementing Change (ERIC) classification [25] into the following categories: ‘Medical reimbursement (utilize financial strategies),’ ‘Clinical support for healthcare professionals (support clinicians/provide interactive assistance),’ ‘Engage patients and their families (engage consumers),’ ‘Practice infrastructure (change infrastructure),’ ‘Train and educate healthcare professionals (train and educate stakeholders),’ ‘Work optimization (adapt and tailor to the context),’ ‘Develop healthcare professional interrelationships (stakeholder interrelationships),’ and ‘Evaluation (use evaluative and iterative strategies).’

The content of each strategy, along with the number and distribution of items per category, was reviewed and discussed by a panel consisting of two physiatrists, one physical therapist, one occupational therapist, and one speech and language pathologist with at least 10 years of experience in cancer rehabilitation. After adjusting the number of items, integrating items, and revising wording, a final consensus was reached. The final version of the implementation strategy list comprised 35 items.

### Surveys on implementation strategies for cancer rehabilitation

In the second survey, barriers to cancer rehabilitation identified as the top three priorities in the first survey round were presented. Next, the final version of the implementation strategy list was provided, and participants rated the effectiveness of each strategy on a 4-point Likert scale.

In the third survey, the results from the second survey round were presented. If consensus on an implementation strategy had not been reached in the previous survey, participants would have been asked to re-rate its effectiveness; since consensus was reached on all items, participants proceeded to evaluate the costs of implementing each strategy and the impact of the strategy on successful implementation using a 4-point scale. Costs included non-monetary expenses, such as labor, space, and time. Successful implementation was defined as ‘establishing the continuous provision of cancer rehabilitation across various medical and care settings, including designated cancer care hospitals, regional medical institutions, convalescent rehabilitation hospitals, hospitals in community-based integrated care, community-based daycare/day services, and home-visit care.’

Between rounds, participants were only provided anonymized summaries of the ranked items. To ensure anonymity, participant profiles, individual responses, and free-form comments were not fed back to the participants themselves. If any item failed to meet the consensus criteria, it was scheduled to be presented to participants again after revising its content or wording.

### Statistical analyses

The median and interquartile range (IQR) values of the 4-point Likert scale to assess the effectiveness of the implementation strategies were calculated, and strategies were prioritized in descending order of median value and the percentage of participants who evaluated the implementation strategies as very effective.

For the cost and impact of the implementation strategies, the mean and standard deviation of 4-point scales were calculated, and an Eisenhower Matrix [26] was created in accordance with the previous research [27], since the mean is also accepted as one of the criteria used for consensus-building [24].

The predefined consensus criteria were as follows:

- The percentage of participants evaluating the strategy as ‘effective’ or ‘very effective’ ≥70% [28].
- For a 4-point scale, IQR ≤ 1 or within the range of mean ± 1.64 SD [24].

Statistical analysis was performed with SPSS version 25.0 (IBM, Armonk, NY).

## Results

### Participants

Of the 100 stakeholders, 86 participants submitted responses in the first survey. One person provided an incomplete response; a final total of 85 participants were included in the analysis. Of the 86 participants in the first survey round, 64 participants, who reported experience with cancer rehabilitation, completed the second survey. Of the 64 participants in the second survey round, 59 participants completed the third survey. The participants’ characteristics are shown in Table 1.

**Table 1:** Participants’ characteristics.

	Survey 1 N = 85	Survey 2 N = 64	Survey 3 N = 59
Sex			
Male	54 (63.5)	42 (65.6)	39 (66.1)
Female	31 (36.5)	22 (34.4)	20 (33.9)
Age (y)			
20–29	0 (0)	0 (0)	0 (0)
30–39	7 (8.2)	6 (9.4)	6 (10.2)
40–49	25 (29.4)	21 (32.8)	19 (32.2)
50–59	39 (45.9)	29 (45.3)	26 (44.1)
60-	14 (16.5)	8 (12.5)	8 (13.6)
Professional category			
Physicians	39 (45.9)	29 (45.3)	28 (47.5)
Physical Medicine and Rehabilitation (PM&R)	17 (20.0)	16 (25.0)	15 (25.4)
Orthopedics	3 (3.5)	2 (3.1)	0 (0)
Surgical Oncology	13 (15.3)	8 (12.5)	9 (15.3)
Medical Oncology	6 (7.1)	3 (4.7)	4 (6.8)
Nurses	11 (12.9)	8 (12.5)	7 (11.9)
Rehabilitation professionals	28 (32.6)	26 (40.6)	23 (39.0)
Physical therapists	16 (18.8)	14 (21.9)	12 (20.3)
Occupational therapists	10 (11.8)	10 (15.6)	9 (15.3)
Speech and language pathologists	2 (2.4)	2 (3.1)	2 (3.4)
Healthcare researchers	3 (3.5)	1 (1.6)	1 (1.7)
Cancer survivors or members of patient support groups	4 (4.7)	0 (0)	0 (0)
Experience in cancer rehabilitation			
Yes	65 (76.5)	64 (100)	59 (100)
No	20 (23.3)	0 (0)	0 (0)
(only for those who have experience in cancer rehabilitation)			
Years of experience in cancer rehabilitation (M ± SD)	16.5 ± 6.6	16.6 ± 6.6	16.6 ± 6.7
Primary affiliation			
Designated cancer hospitals	47 (72.3)	45 (70.3)	40 (67.8)
Other hospitals and clinics providing cancer therapy	6 (9.2)	6 (9.4)	6 (10.2)
Community-based rehabilitation facilities	3 (4.6)	4 (4.7)	3 (5.1)
Not currently involved in clinical practice	9 (13.8)	9 (14.1)	8 (13.6)

In the first survey, responses were obtained from those currently engaged in cancer rehabilitation at the time of response (n = 51) regarding the status of cancer rehabilitation practice (Table 2).

**Table 2:** Status of cancer rehabilitation practice (first survey).

*Cancer rehabilitation classification (Dietz classification)*	n (%)
Preventive	46 (90.2)
Restorative	51 (100)
Supportive	46 (90.2)
Palliative	42 (82.4)
***Cancer status***	
Cancer is stable: life expectancy measured in years	49 (96.1)
life expectancy measured in months	47 (92.2)
life expectancy measured in weeks or days	34 (66.7)
Cancer cured or in complete remission	33 (64.7)
***Implementation Status of Inpatient Rehabilitation***	
Implemented appropriately for all patients in need	6 (11.8)
Implemented for most patients, but not sufficiently for some	36 (70.6)
Implemented only for some patients, not for most	8 (15.7)
Hardly implemented at all	0 (0)
Not implemented	1 (2.0)
***Implementation Status of Outpatient Rehabilitation***	
Implemented appropriately for all patients in need	1 (2.0)
Implemented for most patients, but not sufficiently for some	6 (11.8)
Implemented only for some patients, not for most	22 (43.1)
Hardly implemented at all	15 (29.4)
Not implemented	7 (13.7)

### Barriers to cancer rehabilitation

Table 3 shows the ranking of barriers to cancer rehabilitation based on the results of the first survey. Five items exceeded the agreement threshold (70%): ‘Issues with medical reimbursement (89.2%),’ ‘Insufficient explanation to patients and their families about high-risk conditions (81.5%),’ ‘Shortage of healthcare personnel (80%),’ ‘No opportunities for information sharing as the medical condition worsens (76.9%),’ and ‘Insufficient understanding of the necessity for rehabilitation among patients and their families (73.8%).’

**Table 3:** Barriers to cancer rehabilitation.

*Facilities/Equipment, Healthcare Professionals, and Medical Reimbursement*	n (%)
Issues with medical reimbursement	58 (89.2)
Shortage of healthcare personnel	52 (80)
Lack of knowledge among healthcare professionals	27 (41.5)
Eligible patients are present but not referred to rehabilitation professionals by oncologists	23 (35.4)
Lack of evidence for cancer rehabilitation	15 (23.1)
Lack of awareness of the need for rehabilitation among healthcare professionals	11 (16.9)
Use of medical devices and tubes related to cancer treatment	9 (13.8)
***Medical Collaboration***	
Issues with medical fees	41 (63.1)
Difficulty in timely coordination as the medical condition worsens	40 (61.5)
Lack of venues or opportunities for gathering relevant staff	30 (46.2)
Lack of shared information regarding explanations given to patients and their families	26 (40)
Lack of shared information about cancer treatment plan and life expectancy	25 (38.5)
Unclear points of contact or responsible personnel	19 (29.2)
Lack of shared information regarding recommended physical activity levels	14 (21.5)
***Rehabilitation Goal-setting***	
Differences in goal-setting and treatment plans among healthcare professionals	45 (69.2)
Rapid changes in medical condition prevent timely information sharing	41 (63.1)
Oncologists absent from care conferences or team meetings	39 (60)
Difficulty in outcome prediction in cancer rehabilitation	36 (55.4)
Insufficient information on cancer treatment and life expectancy	34 (52.3)
***Risk Management***	
Insufficient explanation to patients and their families about high-risk conditions	53 (81.5)
No opportunities for information sharing as the medical condition worsens	50 (76.9)
Lack of consistency among healthcare professionals regarding activity level recommendations	40 (61.5)
Inadequate management of physical symptoms	28 (43.1)
Inadequate treatment for high-risk conditions	24 (36.9)
***Communication with cancer patients and their families***	
Insufficient understanding of the necessity for rehabilitation among patients and their families	48 (73.8)
Discrepancies in needs between a patient and his/her family	37 (56.9)
Caregiver burden	32 (49.2)
Difficulty for healthcare professionals in addressing psychological/emotional needs of patients and their families	31 (47.7)
Difficulties in asking patients and their families about their hopes	28 (43.1)
Financial burden on patients and their families	19 (29.2)

**Figure 1:**
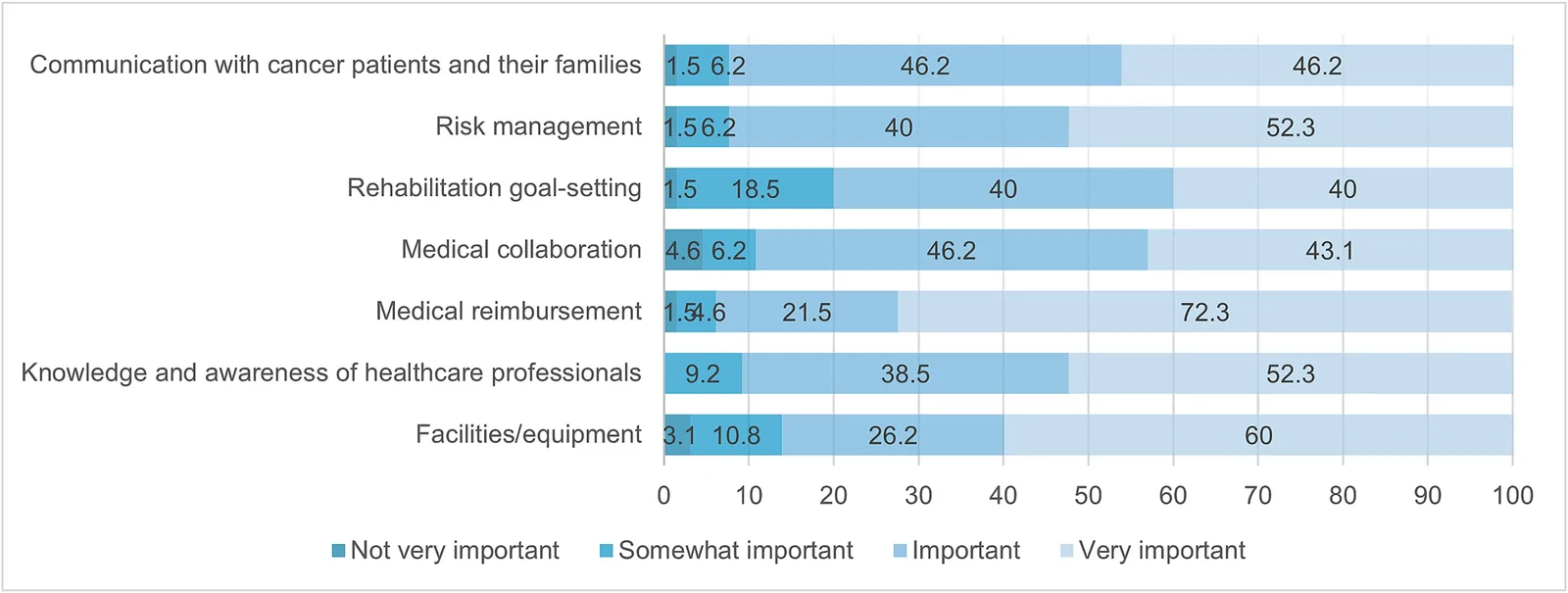
Importance of each domain for cancer rehabilitation practice.

The importance of each domain for cancer rehabilitation practice was prioritized in the order of domains ‘Medical reimbursement’, ‘Risk management’, ‘Communication with cancer patients and their families’, ‘Knowledge and awareness of healthcare professionals’, ‘Medical collaboration’, ‘Facilities/equipment’, and ‘Rehabilitation goal-setting’ (Fig. 1). Regarding risk explanation to cancer patients and families, oncologists and physiatrists were primary providers (38.5% each), followed by rehabilitation professionals (18.5%).

### Implementation strategies for cancer rehabilitation

Table 4 presents the findings from the second survey regarding implementation strategies to minimize barriers to cancer rehabilitation. For all 35 items, >70% of participants rated them as effective (percentage of participants who selected 3 (effective) or 4 (very effective), 70.3–98.4%). The median (IQR) on the 4-point Likert scale was 3.0–4.0 (1). Fourteen items were considered very effective.

**Table 4:** Implementation strategies for cancer rehabilitation.

	Item	Domain	Median	IQR
1	Inclusion of cancer rehabilitation for medical reimbursement in an outpatient setting	Medical reimbursement (utilize financial strategies)	4.00	1
2	Addition of disease rehabilitation to medical reimbursement eligibility in Palliative Care Units	Medical reimbursement (utilize financial strategies)	4.00	1
3	Allocation of rehabilitation specialists in palliative care teams to promote management of physical, psychological, and emotional signs and symptoms	Clinical support for healthcare professionals (support clinicians/provide interactive assistance)	4.00	1
4	Provision of continuous support through health guidance during outpatient care and/or post-discharge home visiting care	Clinical support for healthcare providers (support clinicians/provide interactive assistance)	4.00	1
5	Utilization of mass media to widely promote awareness of the necessity for cancer rehabilitation	Engage patients and their families (engage consumers)	4.00	1
6	Promoting the dissemination of clinical practice guidelines for cancer rehabilitation	Practice infrastructure (change infrastructure)	4.00	1
7	Promoting participation in Cancer Rehabilitation Educational Program for Rehabilitation Teams (CAREER) for healthcare professional training	Train and educate healthcare professionals (train and educate stakeholders)	4.00	1
8	Establishment of undergraduate education systems: Educate and support faculty, integrate cancer rehabilitation into core curricula	Train and educate healthcare professionals (train and educate stakeholders)	4.00	1
9	Establishment of continuing education: Support lifelong learning, establish specialist certification programs	Train and educate healthcare professionals (train and educate stakeholders)	4.00	1
10	Adding rehabilitation specialists to team members to meet requirements for medical fee addition and outpatient management fees for palliative care	Medical reimbursement (utilize financial strategies)	4.00	1
11	Promoting employment of rehabilitation professionals within facilities	Practice infrastructure (change infrastructure)	4.00	1
12	Building and distributing cancer rehabilitation manuals for healthcare professionals	Train and educate healthcare professionals (train and educate stakeholders)	4.00	1
13	Holding study sessions regularly on cancer rehabilitation to enhance staff practical skills	Train and educate healthcare professionals (train and educate stakeholders)	4.00	1
14	Building and distributing educational materials for patients and their families to promote understanding of the necessity for cancer rehabilitation and encourage expression of hopes	Engage patients and their families (engage consumers)	4.00	1
15	(During hospitalization) Encouraging participation of community-based care workers in predischarge conferences to strengthen collaboration	Clinical support for healthcare professionals (support clinicians/provide interactive assistance)	3.50	1
16	Implementation of a system for conveying instructions to ensure consistency in patient activity levels among healthcare professionals	Work optimization (adapt and tailor to the context)	3.50	1
17	Engage healthcare professionals involved in decision-making in cancer care to promote the introduction of cancer rehabilitation	Clinical support for healthcare professionals (support clinicians/provide interactive assistance)	3.00	1
18	Implementation of a system for data sharing between medical and long-term care institutions (e.g. exchanging data with different electronic medical record systems and databases, information sharing apps)	Work optimization (adapt and tailor to the context)	3.00	1
19	Addition of rehabilitation specialists to the list of eligible professions for billing requirements in post-discharge home visiting care	Medical reimbursement (utilize financial strategies)	3.00	1
20	(During hospitalization) Conducting rehabilitation rounds regularly to promote information sharing with attending physicians and ward nurses	Clinical support for healthcare professionals (support clinicians/provide interactive assistance)	3.00	1
21	Establish a nursing collaboration system (nurse-to-nurse coordination) to foster mutual understanding between cancer nursing and rehabilitation nursing	Develop healthcare professional interrelationships (stakeholder interrelationships)	3.00	1
22	Introduction of a fee-for-service plan for anti-cancer drugs in convalescent rehabilitation wards	Medical reimbursement (utilize financial strategies)	3.00	1
23	Building a screening system to identify patients at high risk for cancer rehabilitation	Evaluation (use evaluative and iterative strategies)	3.00	1
24	Organizing a multidisciplinary team specializing in cancer rehabilitation to provide outreach support in community-based healthcare settings	Clinical support for healthcare professionals (support clinicians/provide interactive assistance)	3.00	1
25	Conducting online conferences using information and communication technology (ICT) to encourage multi-occupation participation across institutions	Work optimization (adapt and tailor to the context)	3.00	1
26	Strengthen collaboration with cancer consultation support centers to facilitate access to information	Develop healthcare professional interrelationships (stakeholder interrelationships)	3.00	1
27	Implementation of a system to assess family caregiving burden	Engage patients and their families (engage consumers)	3.00	1
28	Utilization of Personal Health Records (PHRs) to promote self-awareness of cancer-related symptoms and side effects	Engage patients and their families (engage consumers)	3.00	1
29	Introducing patient-reported outcomes (PROs) to promote information sharing across professions	Evaluation (use evaluative and iterative strategies)	3.00	1
30	Establish a system for holding ad hoc conferences as needed according to changes in patient condition or treatment status	Develop healthcare professional interrelationships (stakeholder interrelationships)	3.00	1
31	Allocation of healthcare professionals with cancer rehabilitation experience in convalescent rehabilitation hospitals or hospitals in community-based integrated care	Practice infrastructure (change infrastructure)	3.00	1
32	Promote standardization of information related to cancer rehabilitation by using common assessment tools regardless of disease stage or facility/institution	Evaluation (use evaluative and iterative strategies)	3.00	1
33	Introduce regionally coordinated clinical pathways for cancer rehabilitation to ensure continuity of care	Develop healthcare professional interrelationships (stakeholder interrelationships)	3.00	1
34	Promoting participation in Palliative care Emphasis program on symptom management and Assessment for Continuous medical Education (PEACE) for healthcare professional training	Train and educate healthcare professionals (train and educate stakeholders)	3.00	1
35	Utilization of the International Classification of Functioning, Disability and Health (ICF) as a common language for multidisciplinary collaboration	Evaluation (use evaluative and iterative strategies)	3.00	1

**Figure 2:**
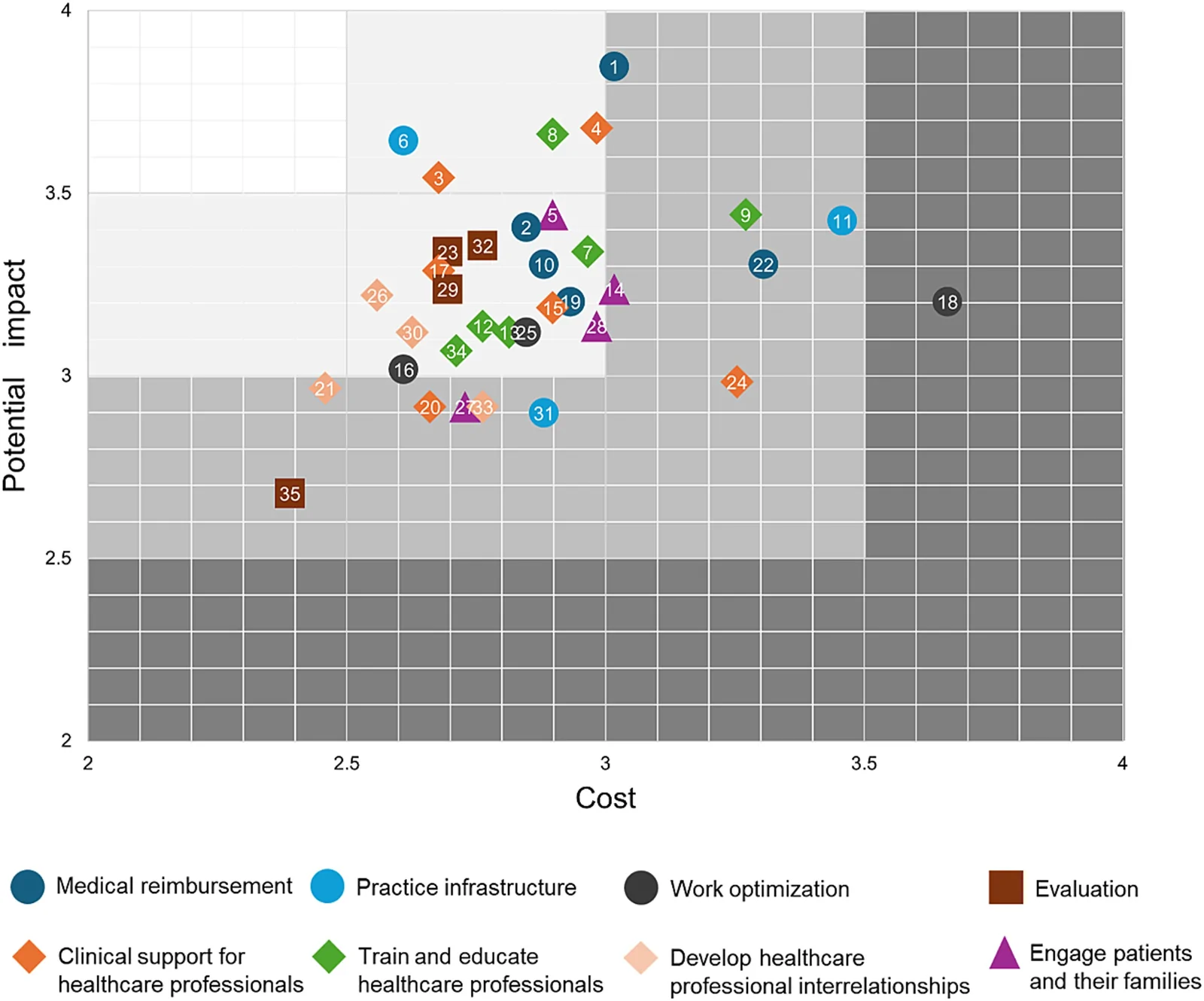
Survey of the priority of the implementation strategies. The vertical axis represents impact, the horizontal axis represents cost, and the average values for each implementation strategy item are plotted. (Both impact and cost range from 2: Slightly low to 4: High.) The background gradient indicates higher priority (higher importance and lower cost) as it becomes brighter.

Since consensus was reached in the second survey round such that all 35 items of the proposed implementation strategy list were deemed valid, the third survey proceeded to assess cost and impact to determine the strategy priorities. The mean (SD) of impact and cost, evaluated on a 4-point scale, were 2.68–3.85 (0.36–0.95) and 2.39–3.66 (0.60–1.07), respectively, with SD within ±1.64. The mean values and SD for impact and cost are listed in Supplementary Table 5. The Eisenhower Matrix is shown in Fig. 2. Considering impact and cost, ‘Engage healthcare professionals involved in decision-making in cancer care to promote the introduction of cancer rehabilitation,’ ‘Building a screening system to identify patients at high risk for cancer rehabilitation,’ ‘Promote standardization of information related to cancer rehabilitation by using common assessment tools regardless of disease stages or facilities/institutions,’ and ‘Strengthen collaboration with cancer consultation support centers to facilitate access to information’ gained higher priorities, in addition to the highly effective strategies mentioned earlier.

The Kruskal-Wallis test (with Bonferroni correction) was used to test for differences in cost and impact by participant profession and care setting (primary affiliation) in an exploratory manner. In the analysis by profession, cost assessments for ‘Implementation of a system to assess family caregiving burden’ and ‘Utilization of Personal Health Records (PHRs) to promote self-awareness of cancer-related symptoms and side effects’ were significantly lower for nurses than for rehabilitation professionals (p < 0.05). In the analysis by care setting, no significant differences in the cost or impact of the implementation strategies were observed.

## Discussion

This study identified implementation strategies and priorities for establishing a seamless cancer rehabilitation provision system from inpatient to home care, based on consensus among diverse stakeholders. By using the Eisenhower Matrix, strategies were successfully categorized not only by effectiveness, but also by feasibility regarding cost, providing a practical roadmap for policymakers and clinical administrators.

Previous studies have reported the current status of cancer rehabilitation at designated cancer care hospitals targeting rehabilitation professionals [29, 30]. However, to the best of our knowledge, this is the first study to target multiple professions and encompass diverse medical and care settings beyond designated cancer care hospitals. It is necessary to assume that the priorities of implementation strategies vary depending on the context, such as different professions and settings. A strength of this study is that it involved diverse stakeholders engaged in cancer rehabilitation across various medical and care settings to establish implementation strategy priorities based on consensus. Successful implementation requires aligning priorities not only with the rehabilitation professionals who provide care, but also with the oncologists and physiatrists who prescribe it; achieving this multidisciplinary consensus is a significant contribution of this study.

In this study, opinion aggregation and consensus formation were conducted using the modified Delphi method. This approach allows for the maintenance of anonymity, controlled feedback, and the recruitment of participants beyond geographical constraints, making it effective for reaching consensus among various stakeholders [23]. A structured questionnaire was used based on a scoping review of prior research to efficiently consolidate opinions, which is an acceptable modification of the Delphi method [23]. Consequently, consensus was reached on all 35 proposed implementation strategies for cancer rehabilitation, which were deemed effective.

The results of the second survey identified several measures as particularly effective, including addressing issues with medical reimbursement, reinforcing undergraduate and postgraduate education systems, strengthening practice infrastructure by increasing rehabilitation personnel, and promoting the dissemination of clinical practice guidelines. The high ranking of educational strategies highlights a critical gap in current oncology training, suggesting that incorporating rehabilitation principles into core curricula is as vital as structural changes.

Furthermore, the priority assessment using the Eisenhower Matrix provided deeper insights. Though structural changes such as reimbursement revision are effective, they are often high-cost or politically difficult to implement immediately. In contrast, the third survey results prioritized ‘reinforcing multidisciplinary collaboration’ and ‘improving access to information.’ These strategies, which focus on process improvement within and between organizations, are relatively lower in cost, but high in impact. This suggests that establishing a seamless cancer rehabilitation provision system requires a dual approach: long-term advocacy for institutional reforms (e.g. reimbursement revisions, human resource cultivation) and immediate efforts to optimize communication and collaboration processes.

### Study limitations

The present study had several limitations. First, the implementation strategies were designed based on Japanese medical and long-term care insurance systems; therefore, generalizability to other countries requires caution. Second, the Delphi method may over-represent participants with a high interest in the topic, and this research methodology could carry the potential for participant bias. Cancer survivors and members of patient support groups were invited to participate in this study. Their cooperation was obtained in the first survey round, and the perspectives of cancer survivors were reflected to some extent in the identification of barriers to cancer rehabilitation. However, subsequent survey rounds were unable to sufficiently incorporate their perspective, which is a limitation of this study. Therefore, future research should increase the sample size of patient support groups to better reflect their opinions. Finally, the results regarding priority order may vary depending on participant distribution, such as by profession or care setting. In this study, an exploratory analysis by profession and affiliation was conducted; however, differences emerged only in the cost assessment of a few strategies by profession. More specifically, for strategies engaging patients and their families, the barriers tended to be lower for nurses and higher for rehabilitation professionals. However, for other strategies, no significant differences were found, suggesting that these implementation strategies could be widely utilized in cancer rehabilitation practice in Japan.

## Conclusions

Implementation strategies and priorities for cancer rehabilitation were identified based on expert consensus. The findings are expected to bridge the gap between evidence and clinical practice in cancer rehabilitation in Japan, promoting the provision of cancer rehabilitation not only at designated cancer care hospitals, but also at regional medical institutions and in community-based home care.

## Supplementary Material

Supplementary_Tables_JJCO-26-0093_revision_submit_hyag085

## Data Availability

Not applicable.
