# Supplementary material for: Implementation strategies for cancer rehabilitation in clinical practice: a Delphi study
Source: Jpn J Clin Oncol. 2026 Jun 10;56(9):1052–61. doi: 10.1093/jjco/hyag085 (PMC13556456; doi:10.1093/jjco/hyag085)
Supplement: Supplementary file 1 [file supplementary_tables_jjco-26-0093_revision_submit_hyag085.docx]

**Supplementary Table 1. Members of the Cancer Rehabilitation and Lymphedema Care Network Consortium**

| Organization | References |
| --- | --- |
| - Geriatric Health Services Facility in Japan - Japan Visiting Nursing Foundation - Japan Association for Day Care - Japan Care Manager Association - Japan Association of Rehabilitation Hospital and Institution - Cancer Fitness - NPO Lymphcafe - Lymphedema Network Japan - Japanese Association of Social Workers in Health Service - Japan Society of Gynecologic Oncology - Japanese Physical Therapy Association - Japanese Association of Rehabilitation Medicine - Japanese Society of Cancer Nursing - Japanese Association of Supportive Care in Cancer - Japanese Society of Physical Therapy for Oncology and Lymphedema - Japan Society of Plastic and Reconstructive Surgery - Japanese Association of Speech-Language-Hearing Therapists - Japanese Association of Occupational Therapists - Japanese Society of Phlebology - Japanese Society of Community-based Comprehensive Physical Therapy - Japanese Breast Cancer Society - Japanese Lymphedema Society - Japanese Society for Lymphedema Therapy - Japanese Society for Palliative Medicine - Japan Rehabilitation Nurse Association - Japanese Society of Lymphology | https://www.roken.or.jp/  https://www.jvnf.or.jp/  https://day-care.jp/  https://www.jcma.or.jp/  https://www.rehakyoh.jp/  https://cancerfitness.jp/  https://lymphcafe.org/  https://lymnet.jp/  https://www.jaswhs.or.jp/  https://jsgo.or.jp/en/  https://www.japanpt.or.jp/english/  https://www.jarm.or.jp/english/  https://jscn.or.jp/en/  http://jascc.jp/en/home/  https://www.jspt.or.jp/jspto/  https://jsprs.or.jp/english/  https://www.japanslht.or.jp/english/  https://www.jaot.or.jp/en/  https://js-phlebology.jp/wpenglish/  https://www.jsccpt.jp/  https://www.jbcs.gr.jp/modules/English/index.php?content_id=1  https://www.js-lymphedema.org/  https://jslt.site/  https://www.jspm.ne.jp/english/  https://www.jrna.or.jp/  https://lymphology.umin.jp/ |

**Supplementary Table 2. Questionnaire, Survey 1**

| Item Number | Questions | Response |
| --- | --- | --- |
| *Section 1: Demographics* | | |
| Q1 | Please indicate your age. | 1. 20-29  2. 30-39  3. 40-49  4. 50-59  5. 60- |
| Q2 | Please indicate your sex. | 1. Female  2. Male  3. Prefer not to answer |
| Q3 | Please indicate your current professional category. | 1. Physician  2. Nurse  3. Physical therapist  4. Occupational therapist  5. Speech and language pathologist  6. Healthcare researcher  7. Cancer survivor or member of a patient support group  8. Other |
| Q3-1 | If you selected “1. Physician,” please enter your specialty. | Free text |
| Q4 | Do you have experience in cancer rehabilitation? | 1. Yes  2. No |
| Q4-1 | If you selected “1. Yes,” please enter your total years of experience. | Number of years |
| Q5 | Please indicate your primary location of practice. | 1. Designated cancer hospitals  2. Other hospitals and clinics providing cancer therapy  3. Hospitals and clinics not providing cancer care (e.g., convalescent rehabilitation hospitals)  4. Community-based rehabilitation facilities  5. Other  6. Not currently involved in clinical practice |
| **Q6** | Does your current workplace provide rehabilitation for cancer patients? (This indicates cases where cancer patients rehabilitation fees and/or disease rehabilitation fees are billed for cancer patients.) | 1. Yes (If yes, proceed to Q7.)  2. No (If no, proceed to Q11.) |
| *Section 2: Status of Cancer Rehabilitation Practice* | | |
| Q7 | Please indicate cancer classification (Dietz classification) for patients receiving rehabilitation. Please select all that apply. | 1. Preventive  2. Restorative  3. Supportive  4. Palliative |
| Q8 | Please indicate the cancer status of patients receiving cancer rehabilitation. Please select all that apply. | 1. Cancer is stable: life expectancy measured in years  2. Cancer is stable: life expectancy measured in months  3. Cancer is stable: life expectancy measured in weeks or days  4. Cancer cured or in complete remission |
| Q9 | Is inpatient rehabilitation for cancer patients appropriately implemented at your facility? Please select the statement that best reflects your opinion. | 1. Implemented appropriately for all patients in need 2. Implemented for most patients, but not sufficiently for some 3. Implemented only for some patients, not for most 4. Hardly implemented at all 5. Not implemented |
| Q10 | Is outpatient rehabilitation for cancer patients appropriately implemented at your facility? Please select the statement that best reflects your opinion. | 1. Implemented appropriately for all patients in need 2. Implemented for most patients, but not sufficiently for some 3. Implemented only for some patients, not for most 4. Hardly implemented at all 5. Not implemented |
| *Section 3: Status of Cancer Rehabilitation Practice*  Based on your clinical experience, please indicate the barriers to cancer rehabilitation | | |
| Q11 | Regarding communications with cancer patients and their families, please indicate the reasons that constitute barriers to providing cancer rehabilitation for you.  Please select up to three statements that best reflect your opinion.  Free response comments are not counted. | 1. Insufficient understanding of the necessity for rehabilitation among patients and their families 2. Difficulty for healthcare professionals in addressing psychological/emotional needs of patients and their families 3. Difficulties in asking patients and their families about their hopes 4. Discrepancies in needs between a patient and his/her family 5. Financial burden on patients and their families 6. Caregiver burden 7. Other (free text) |
| Q12 | Regarding risk management, please indicate the reasons that constitute barriers to providing cancer rehabilitation for you.  Please select up to three statements that best reflect your opinion.  Free response comments are not counted. | 1. Inadequate treatment for high-risk conditions  2. Insufficient explanation to patients and their families about high-risk conditions  3. Inadequate management of physical symptoms  4. Lack of consistency among healthcare professionals regarding activity level recommendations  5. No opportunities for information sharing as the medical condition worsens  6. Other (free text) |
| Q13 | At your facility, who explains the risks to patients and their families when starting rehabilitation treatment? Please select the statement that best reflects your opinion. If your current facility does not provide cancer rehabilitation, please select the most applicable option based on your previous clinical experience. | 1. Oncologists 2. Primary care physicians 3. Physiatrists 4. Nurses 5. Rehabilitation professionals 6. Other (free text) |
| Q14 | Regarding rehabilitation goal-setting, please indicate the reasons that constitute barriers to providing cancer rehabilitation for you.  Please select up to three statements that best reflect your opinion.  Free response comments are not counted. | 1. Insufficient information on cancer treatment and life expectancy  2. Difficulty in outcome prediction in cancer rehabilitation  3. Rapid changes in medical condition prevent timely information sharing  4. Oncologists absent from care conferences or team meetings  5. Differences in goal-setting and treatment plans among healthcare professionals  6. Other (free text) |
| Q15 | Regarding medical collaboration, please indicate the reasons that constitute barriers to providing cancer rehabilitation for you.  Please select up to three statements that best reflect your opinion.  Free response comments are not counted. | 1. Lack of venues or opportunities for gathering relevant staff  2. Difficulty in timely coordination as the medical condition worsens  3. Lack of shared information about cancer treatment plan and life expectancy  4. Lack of shared information regarding explanations given to patients and their families  5. Lack of shared information regarding recommended physical activity levels  6. Unclear points of contact or responsible personnel  7. Issues with medical fees  8. Other (free text) |
| Q16 | Regarding facilities/equipment, healthcare professionals, and medical reimbursement, please indicate the reasons that constitute barriers to providing cancer rehabilitation for you.  Please select up to three statements that best reflect your opinion.  Free response comments are not counted. | 1. Use of medical devices and tubes related to cancer treatment  2. Shortage of healthcare personnel  3. Lack of knowledge among healthcare professionals  4. Eligible patients are present but not referred to rehabilitation professionals from oncologists  5. Lack of evidence for cancer rehabilitation  6. Lack of awareness of the need for rehabilitation among healthcare professionals  7. Issues with medical reimbursement |
| Q17 | Which areas are important for optimally implementing cancer rehabilitation? Please select the option that best reflects your opinion.  A. Communication with cancer patients and their families  B. Risk management  C. Rehabilitation goal-setting  D. Medical collaboration  E. Facilities/equipment  F. Knowledge and awareness of healthcare professionals  G. Medical reimbursement | 1. Not very important  2. Somewhat important  3. Important  4. Very important |

**Supplementary Table 3. Questionnaire, Survey 2**

| Item Number | Questions | Response |
| --- | --- | --- |
| Please refer to each strategy in the implementation strategy list (35 items). | | |
| Q1-35 | How effective do you consider each strategy to be in minimizing barriers to cancer rehabilitation?  Please select the option that best reflects your opinion. | 1. Ineffective  2. Not very effective  3. Effective  4. Very effective |

**Supplementary Table 4. Questionnaire, Survey 3**

| Item Number | Questions | Response |
| --- | --- | --- |
| Q1-35 | (Only strategies for which consensus on effectiveness was reached in the second survey are presented.)  How important do you consider each strategy to be for the successful implementation of cancer rehabilitation?  Please select the option that best reflects your opinion. | 1. Low  2. Slightly Low  3. Slightly High  4. High |
| Q1-35 | How significant do you consider the costs required to implement each strategy to be?  Please select the option that best reflects your opinion.  “Costs” include non-monetary expenses such as labor, space, and time. | 1. Low  2. Slightly Low  3. Slightly High  4. High |

**Supplementary Table 5. Importance and Cost of Implementation Strategies**

| **Implementation strategies** | | **Importance, mean** | **Importance, SD** | **Cost, mean** | **Cost,  SD** |
| --- | --- | --- | --- | --- | --- |
| 1 | Inclusion of cancer rehabilitation for medical reimbursement in an outpatient setting | 3.85 | 0.36 | 3.02 | 0.83 |
| 4 | Provision of continuous support through health guidance during outpatient care and/or post-discharge home visiting care | 3.68 | 0.50 | 2.98 | 0.81 |
| 8 | Establishment of undergraduate education systems: Educate and support faculty, integrate cancer rehabilitation into core curricula | 3.66 | 0.54 | 2.90 | 0.86 |
| 6 | Promoting the dissemination of clinical practice guidelines for cancer rehabilitation | 3.64 | 0.55 | 2.61 | 0.94 |
| 3 | Allocation of rehabilitation specialists in palliative care teams to promote management of physical, psychological, and emotional signs and symptoms | 3.54 | 0.62 | 2.68 | 0.87 |
| 5 | Utilization of mass media to widely promote awareness of the necessity for cancer rehabilitation | 3.44 | 0.77 | 2.90 | 1.07 |
| 9 | Establishment of continuing education: Support lifelong learning, establish specialist certification programs | 3.44 | 0.65 | 3.27 | 0.84 |
| 11 | Promoting employment of rehabilitation professionals within facilities | 3.42 | 0.69 | 3.46 | 0.81 |
| 2 | Addition of disease rehabilitation to medical reimbursement eligibility in Palliative Care Units | 3.41 | 0.74 | 2.85 | 0.90 |
| 32 | Promote standardization of information related to cancer rehabilitation by using common assessment tools regardless of disease stage or facility/institution | 3.36 | 0.73 | 2.76 | 0.87 |
| 7 | Promoting participation in Cancer Rehabilitation Educational Program for Rehabilitation Teams (CAREER) for healthcare professional training | 3.34 | 0.70 | 2.97 | 0.74 |
| 23 | Building a screening system to identify patients at high risk for cancer rehabilitation | 3.34 | 0.70 | 2.70 | 0.94 |
| 10 | Adding rehabilitation specialists to team members to meet requirements for medical fee addition and outpatient management fees for palliative care | 3.31 | 0.79 | 2.88 | 0.83 |
| 22 | Introduction of a fee-for-service plan for anti-cancer drugs in convalescent rehabilitation wards | 3.31 | 0.85 | 3.31 | 0.79 |
| 17 | Engage healthcare professionals involved in decision-making in cancer care to promote the introduction of cancer rehabilitation | 3.29 | 0.67 | 2.68 | 0.75 |
| 14 | Building and distributing educational materials for patients and their families to promote understanding of the necessity for cancer rehabilitation and encourage expression of hopes | 3.24 | 0.70 | 3.02 | 0.73 |
| 29 | Introducing patient-reported outcomes (PROs) to promote information sharing across professions | 3.24 | 0.72 | 2.70 | 0.77 |
| 26 | Strengthen collaboration with cancer consultation support centers to facilitate access to information. | 3.22 | 0.76 | 2.56 | 0.83 |
| 18 | Implementation of a system for data sharing between medical and long-term care institutions (e.g., exchanging data with different electronic medical record systems and databases, information sharing apps) | 3.20 | 0.80 | 3.66 | 0.60 |
| 19 | Addition of rehabilitation specialists to the list of eligible professions for billing requirements in post-discharge home visiting care | 3.20 | 0.86 | 2.93 | 0.78 |
| 15 | (During hospitalization) Encouraging participation of community-based care workers in predischarge conferences to strengthen collaboration | 3.19 | 0.65 | 2.90 | 0.84 |
| 12 | Building and distributing cancer rehabilitation manuals for healthcare professionals | 3.14 | 0.89 | 2.76 | 0.74 |
| 28 | Utilization of Personal Health Records (PHRs) to promote self-awareness of cancer-related symptoms and side effects | 3.14 | 0.81 | 2.98 | 0.73 |
| 13 | Holding study sessions regularly on cancer rehabilitation to enhance staff practical skills | 3.12 | 0.72 | 2.81 | 0.85 |
| 25 | Conducting online conferences using information and communication technology (ICT) to encourage multi-occupation participation across institutions | 3.12 | 0.78 | 2.85 | 0.86 |
| 30 | Establish a system for holding ad hoc conferences as needed according to changes in patient condition or treatment status | 3.12 | 0.72 | 2.63 | 0.84 |
| 34 | Promoting participation in Palliative care Emphasis program on symptom management and Assessment for Continuous medical Education (PEACE) for healthcare professional training | 3.07 | 0.63 | 2.71 | 0.82 |
| 16 | Implementation of a system for conveying instructions to ensure consistency in patient activity levels among healthcare professionals | 3.02 | 0.83 | 2.61 | 0.90 |
| 24 | Organizing a multidisciplinary team specializing in cancer rehabilitation to provide outreach support in community-based healthcare settings | 2.98 | 0.85 | 3.25 | 0.88 |
| 21 | Establish a nursing collaboration system (nurse-to-nurse coordination) to foster mutual understanding between cancer nursing and rehabilitation nursing | 2.97 | 0.86 | 2.46 | 0.81 |
| 20 | (During hospitalization) Conducting rehabilitation rounds regularly to promote information sharing with attending physicians and ward nurses | 2.92 | 0.87 | 2.66 | 0.91 |
| 27 | Implementation of a system to assess family caregiving burden | 2.92 | 0.85 | 2.73 | 0.78 |
| 33 | Introduce regionally coordinated clinical pathways for cancer rehabilitation to ensure continuity of care | 2.92 | 0.81 | 2.76 | 0.93 |
| 31 | Allocation of healthcare professionals with cancer rehabilitation experience in convalescent rehabilitation hospitals or hospitals in community-based integrated care | 2.90 | 0.75 | 2.88 | 0.80 |
| 35 | Utilization of the International Classification of Functioning, Disability and Health (ICF) as a common language for multidisciplinary collaboration | 2.68 | 0.95 | 2.39 | 0.98 |
